# Educational approaches to the relationship between cervical adjusting and risk of cervical artery dissection; a survey of chiropractic faculty

**DOI:** 10.1186/s12998-026-00675-7

**Published:** 2026-09-02

**Authors:** Chad Lambert, Kathryn Ross, Christine Major, Kara Burnham, Shawn Hatch, Brian Anderson

**Affiliations:** 1 University of Western States, 8000 NE Tillamook St Portland, OR Portland, 97213 USA; 2 Department of Community Health Services and Rehabilitation Science, University of Pittsburgh, 3396 Fifth Avenue, Pittsburgh, PA 15213 USA

**Keywords:** Manipulation, spinal, Questionnaires and Surveys, Education, Risk management, Vertebral artery dissection

## Abstract

**Background:**

Cervical artery dissection (CeAD) remains a rare but clinically important condition that has generated longstanding debate regarding its potential association with cervical spinal manipulative therapy (cSMT). Although biomechanical and epidemiologic studies have examined this relationship, little is known about how chiropractic educational programs address CeAD or how individual faculty member’s perceptions shape instructional practices. This study surveyed chiropractic faculty members who actively teach cervical manipulation to understand how educators conceptualize CeAD risk and incorporate safety considerations into curricula.

**Methods:**

An anonymous twelve-item electronic survey was distributed via Qualtrics to institutional gatekeepers from 28 English-speaking chiropractic programs between February and March 2025. Gatekeepers then distributed the survey to instructors with hands-on experience teaching cervical manipulation within the prior five years. Survey items assessed teaching setting, beliefs about the cSMT–CeAD relationship, and whether perceived risk influenced screening procedures and technique modification. Quantitative data were analyzed using descriptive statistics, Pearson’s chi-square tests, and Fisher’s exact test; qualitative free-text responses underwent keyword extraction and keyword-based thematic analysis.

**Results:**

Individual faculty members from 17 of the 28 invited chiropractic programs participated (61% program response rate), yielding 103 responses with 85 meeting inclusion criteria (46 classroom, 38 clinical, 1 undisclosed). Teaching setting was significantly associated with faculty perspectives of CeAD causation (p = 0.013); classroom faculty more frequently characterized the cSMT – CeAD relationship as associational (73% vs. 27%), while clinical faculty more frequently endorsed a causal relationship mediated by modifiable risk factors (63% vs. 33%). Most respondents (72%) reported that CeAD risk influenced their instructional approach, primarily through pretreatment screening and technique modification. Qualitative analysis of free-text responses (n = 19) identified four themes: Clinical Assessment & Management (89.5% of responses), Risk & Safety (52.6%), Education & Training (31.6%), and Evidence Base (21.1%).

**Conclusions:**

Teaching setting significantly influenced how chiropractic faculty members conceptualize and communicate CeAD risk. Findings reveal opportunities to strengthen evidence-aligned patient safety education and establish instructional guidance across chiropractic programs. The gatekeeper-led sampling approach limits individual level response rate calculation and generalizability.

**Supplementary Information:**

The online version contains supplementary material available at https://doi.org/10.1186/s12998-026-00675-7.

## Background

Early biomechanical studies suggested that upper cervical manipulation (cSMT) involving rotation may impart mechanical stress on the vertebral artery, potentially precipitating cervical artery dissection (CeAD) [1, 2]. CeAD encompasses both internal carotid artery dissections and vertebral artery dissections [3]. However, the present study focuses specifically on vertebral artery dissection, given its closer anatomical proximity to cervical manipulative contact points and its more prominent role in the cSMT safety literature. The V3 segment of the vertebral artery, travelling through the transverse foramen of C2, through the C1 transverse foramen and then crossing the posterior arch of C1, is frequently implicated in traumatic vertebral artery dissections [3]. This segment may experience tensile forces during neck rotation, temporarily decreasing vascular flow [4]. However, more recent studies demonstrate greater tensile forces occur during normal range of motion than during cSMT [5] and no significant difference in blood flow through the vertebral arteries was found between neutral head position, passive cervical rotation, and cSMT [6]. Further, Gorrell et al. [7] found that while cSMT does produce vertebral artery elongation, the forces generated were insufficient to damage otherwise healthy vascular structures. It is important to note that this study was performed on unembalmed cadavers with significant tissue removed by dissection, limiting generalizability.

Despite numerous reports documenting cases of CeAD temporally associated with cSMT, the literature infrequently reports adequate descriptive treatment details to further our understanding of the relationship between cSMT and CeAD [8]. Grainger [9] stated that dissection is a result of “extension and rotation of the neck beyond the physiological range of motion.” Rotation and extension have been cited as potential risk factors in existing literature both supporting and questioning a causal link between cSMT and CeAD [3]. This ongoing debate has likely influenced practice behaviors among chiropractors, yet it remains unclear whether educational institutions have integrated this evolving evidence into their curricula or modified instructional delivery of cervical manipulation accordingly. In addition, a gap exists in the chiropractic literature regarding the teaching of students about CeAD; however, this has been explored in other professions [10]. 

Currently, no published research has evaluated whether chiropractic programs adapt cSMT instruction in response to CeAD risk factors or broader patient safety concerns [11]. Little is known about how curricula address CeAD when teaching cervical manipulative procedures, or whether individual faculty members independently modify techniques to mitigate perceived risks. The objective of this study was to survey chiropractic faculty members who actively teach cervical manipulation to examine how perceived CeAD-related risks influence instructional approaches in both classroom and clinical settings, and to characterize the degree to which individual faculty’s beliefs about the cSMT-CeAD relationship shape pedagogical decision-making.

## Methods

The study employed a cross-sectional survey design to examine how chiropractic individual faculty members perceive the relationship between cSMT and CeAD, and how those perceptions influence instructional practices. Reporting follows the Consensus-Based Checklist for Reporting of Survey Studies (CROSS, Appendix B). This project was reviewed by the lead author’s institutional review board (IRB) and deemed exempt from full review due to the anonymous, deidentified nature of data collection. No financial or other incentives were offered for participation.

### Participants

Individual faculty members from 28 English-speaking chiropractic programs in the United States, Canada, Australia, and the United Kingdom were invited to complete an anonymous electronic survey between February and March 2025. Initial institutional contacts—typically deans or research directors (*n* = 28)— were identified through program website searches. Contacts from the United States, Australia, and the United Kingdom then distributed the survey to faculty members within their institutions who had hands-on experience teaching cervical manipulation within the prior five years. This recruitment approach constitutes a convenience sample facilitated by institutional gatekeepers.

### Survey instrument

Survey items were informed by literature published within the preceding ten years on the cSMT – CeAD relationship, as well as the lead authors instructional expertise in cervical assessment and technique. To assess face validity, four chiropractic faculty members with subject-matter expertise in cervical spine assessment and manipulative technique reviewed the draft instrument. Reviewer feedback informed revisions to item wording, response option clarity, and overall question order; no items were removed, but several response stems were reworded to reduce ambiguity. An IRB administrator independently evaluated the survey for readability and adherence to human subjects’ requirements. The full survey instrument is provided in Appendix A.

### Quantitative data analysis

Descriptive statistics, including frequencies, percentages, and totals, were used to summarize responses across all survey items. Bivariate associations between related responses were examined using cross-tabulations and Pearson’s chi-square tests, excluding cases with missing responses in either variable. Responses to the item assessing expert opinion on the cSMT–CeAD relationship (Q4) were recoded into three mutually exclusive categories for bivariate analysis, grouped by their shared instructional implications. The *Association* category combined “never causal” and “rarely causal” responses, as both reflect a belief that cSMT poses negligible or no causal risk and does not motivate modification of pretreatment or technique instruction. The *Causal Link* combined “modifiable by history and examination” and “modifiable by technique” responses, reflecting a belief that cSMT can cause CeAD and that practitioner behavior can reduce such risk. The *Other category* combined “no association,” “causal but non-modifiable,” and “other” responses; “causal but non-modifiable” was placed in this category as it implies no actionable risk reduction. Respondents who did not answer Q4 were retained as a separate No Response category in descriptive displays but excluded from chi-square significance testing. Associations between this recoded belief variable and teaching setting, pretreatment instruction, and technique modification practices were examined using Pearson’s chi-square tests, with p-values reported throughout. Resource endorsement (Q10) was compared across teaching setting and belief category using Fisher’s exact test for individual resources and the Mann-Whitney U test for total resource count; respondents selecting “Other” as their only response or not answering Q10 were excluded (*n* = 9), yielding an analytic subsample of *n* = 76. Statistical analyses were conducted using Python (version 3.12).

### Qualitative data analysis

Free-text responses to the final survey question – “Is there anything else you would like to describe regarding your approach to teaching chiropractic technique and cervical artery dissection?” – were analyzed using keyword extraction and topic modeling techniques to provide contextual texture for quantitative findings rather than to support independent conclusions. Given the small number of responses, qualitative results should be interpreted as exploratory and hypothesis-generating. A multi-level n-gram frequency analysis (uni-, bi-, and trigrams) identified common linguistic patterns after removing stop words using regular expression coding [12]. In parallel, keyword-based thematic analysis was conducted using a manually developed dictionary of themes and associated keywords. Each response was scored by keyword frequency to quantify thematic presence, and this keyword-theme mapping provided the framework for thematic development. Response lengths were summarized by word and character counts, and exploratory visualizations included bar charts of the most frequent words and thematic categories and a histogram of word count distributions [13]. 

## Results

### Participants

Of the 28 chiropractic programs contacted via email, two programs declined participation, and nine did not respond, yielding individual faculty survey responses (*n* = 103) from 17 programs (61% institutional response rate). Because the survey was distributed by institutional gatekeepers rather than directly to individual faculty members, total count of individual faculty who received the survey invitation cannot be determined, precluding calculation of an individual-level participation rate.

After data cleaning, 18 submissions were excluded due to ineligible or missing responses to the screener, producing a final analytic sample of 85 respondents. Of these, 46 (54%) reported teaching primarily in classroom settings and 38 (45%) in clinical settings; one preferred not to disclose their teaching role. Most respondents (72%, *n* = 56) indicated that CeAD-related concerns influenced their instructional approach. Among those whose instruction was influenced by CeAD risk, minimizing the combination of rotation and extension was the most common technique modification, whether for the upper cervical spine (75%, *n* = 12) or entire cervical spine (69%, *n* = 52). Full response distributions are presented in Table 1.

**Table 1:** Survey Responses of Chiropractic Faculty Regarding Cervical Adjusting and Cervical Artery Dissection (*N* = 85)

Question	Response	*n* (%)
**Q1: Have you taught some form of hands-on cervical manipulation or adjusting in the past 5 years at a college or university? (** ***N*** ** = 98 responded)**		
	Yes (eligible; analytic sample)	85 (87%)
	No (ineligible; excluded)	13 (13%)
**Q2: Please select the setting in which you teach cervical adjusting.** (*n* = 85)		
	Classroom setting primarily	46 (54%)
	Clinical setting primarily	38 (45%)
	Prefer not to answer	1 (1%)
**Q3: Is the reported correlation between cervical adjusting and cervical artery dissection a subject you discuss when teaching?** (*n* = 84)		
	Yes	71 (84%)
	No	13 (15%)
	No response	1 (1%)
**Q4: Which of the following phrases best describes your expert opinion on this association?** (*n* = 68)		
	No association between cervical adjustments and CeAD	4 (6%)
	Association but it is never causal	4 (6%)
	Association but it is rarely causal	26 (38%)
	Causal link modifiable with history and exam	19 (28%)
	Causal link – non-modifiable	1 (1%)
	Causal link influenced by SMT technique	7 (10%)
	Other	7 (10%)
	No response (*N* = 85)ᵃ	17 (20%)
**Q5: Does the risk of CeAD affect how you teach pretreatment procedures prior to adjusting?** (*n* = 78)		
	Yes	56 (72%)
	No	22 (28%)
	No response (*N* = 85)ᵃ	7 (8%)
**Q6: Which of the following descriptions most closely aligns with how you teach pretreatment procedures?** (*n* = 55)		
	Among *n* = 56 who reported CeAD affects pretreatment teaching (Q5); percentages reflect this subgroup	
	Questions to assess likelihood of CeAD in progress	18 (33%)
	Questions assessing likelihood of underlying condition increasing CeAD risk when adjusted	21 (38%)
	Questions/exams assessing likelihood of underlying condition for which SMT may be erroneously blamed	12 (22%)
	Other	4 (7%)
	Did not endorse specific pretreatment approach (full sample, *N* = 85)ᵃ	30 (35%)
**Q7: If you believe modifications to cervical adjusting technique may (or may not) influence the risk of CeAD**,** which resource(s) most strongly influence that belief? (Select all that apply;***n* = 76)		
	Systematic reviews and meta-analyses of the literature	44 (58%)
	Personal/clinical experience	40 (53%)
	Anatomic/cadaveric studies in the scientific literature	34 (45%)
	Case reports in the scientific literature	31 (41%)
	Clinical experience of colleagues	29 (38%)
	Expert opinion of chiropractic educators	28 (37%)
	Narrative reviews of the literature	12 (16%)
	Other	4 (5%)
**Q8: Does the risk of CeAD affect how you teach adjusting technique of the upper cervical spine only or the entire cervical spine?** (*n* = 77)		
	Entire cervical spine	52 (68%)
	Neither	13 (17%)
	Upper cervical spine only	12 (16%)
	No response (*N* = 85)ᵃ	8 (9%)
**Q9: Which of the following most closely aligns with the modification(s) to upper cervical adjusting technique you use when you teach?**		
	Among *n* = 12 who reported upper cervical instruction affected (Q8); percentages reflect this subgroup	
	Minimizing rotation AND extension in the upper cervical spine	9 (75%)
	Minimizing rotation in the upper cervical spine	1 (8%)
	Minimizing lateral flexion/bending AND extension	1 (8%)
	Other	1 (8%)
	Did not endorse upper cervical modification (full sample, *N* = 85)ᵃ	73 (86%)
**Q10: Which of the following most closely aligns with the modification(s) to entire cervical spine adjusting technique you use when you teach?**		
	Among *n* = 52 who reported entire cervical spine instruction affected (Q8); percentages reflect this subgroup	
	Minimizing rotation AND extension in the cervical spine	36 (69%)
	Minimizing rotation only	8 (15%)
	Minimizing rotation AND lateral flexion/bending	3 (6%)
	Minimizing lateral flexion/bending AND extension	2 (4%)
	Minimizing extension only	1 (2%)
	Other	2 (4%)
	Did not endorse full cervical modification (full sample, *N* = 85)ᵃ	33 (39%)
**Q11: Does the risk of CeAD affect how you teach adjusting technique of the cervicothoracic spine?** (*n* = 76)		
	No	45 (59%)
	Yes	31 (41%)
	No response (*N* = 85)ᵃ	9 (11%)
**Q12: Which of the following most closely aligns with the modification(s) to cervicothoracic adjusting technique you use when you teach?**		
	Among *n* = 31 who reported cervicothoracic instruction affected (Q11); percentages reflect this subgroup	
	Minimizing the combination of rotation AND extension	19 (61%)
	Other	5 (16%)
	Minimizing rotation only	4 (13%)
	Minimizing the combination of lateral flexion/bending AND extension	2 (6%)
	Minimizing the combination of rotation AND lateral flexion/bending	1 (3%)
	Did not endorse cervicothoracic modification (full sample, *N* = 85)ᵃ	54 (64%)

**a** Full-sample non-response row: denominator is *N* = 85 (all eligible respondents); presented to contextualize overall prevalence of the subgroup finding. Italicized rows use a different denominator than indented response rows above

Abbreviations: CeAD, cervical artery dissection; SMT, spinal manipulative therapy

### Quantitative results

Table 1 summarizes the distribution of responses across all survey items, including faculty roles, beliefs about the CeAD – cSMT association, and reported influences on instructional practices. Responses to the item assessing expert opinion on the CeAD – cSMT relationship were recoded into three mutually exclusive categories: *Association* (e.g., “rarely” or “never causal”), *Causal Link* (e.g., “modifiable risk”), and *Other* (e.g., “no association”, “causal but non-modifiable”, or “Other”). No significant associations were observed between teaching setting and most categorical variables; however, key differences emerged regarding perceived causation and its instructional influence. 

Among 85 respondents, 35% (*n* = 30) characterized the cSMT–CeAD relationship as associational, 32% (*n* = 27) endorsement of causality, and 13% (*n* = 11) selected other; 17 provided no response (Table 2). Teaching setting was significantly associated with faculty members’ perceptions of causation (*p* = 0.013): individual faculty endorsing an associational relationship predominantly taught in classroom settings (73%), while those endorsing a causal link more often taught in clinical settings (63%). Both groups reported CeAD risk influenced pretreatment screening instruction at similarly high rates (83% and 85%, respectively), whereas the “other” group reported substantially lower influence (36%; *p* = 0.003). Educators endorsing an association more frequently reported CeAD risk influenced cervicothoracic SMT instruction (57% vs. 41% causal; *p* = 0.005), while reported influence on upper cervical spine instruction was uniformly low across groups (17% association vs. 7% causal; *p* < 0.001).

**Table 2:** Faculty beliefs about the cSMT–CeAD relationship by teaching setting (*N* = 85)

	Association (*n* = 30)	Causal Link (*n* = 27)	Other (*n* = 11)	No Response (*n* = 17)	Total (*n* = 85)
Teaching setting*					
Classroom	22 (73%)	9 (33%)	5 (45%)	10 (59%)	46 (54%)
Clinic	8 (27%)	17 (63%)	6 (55%)	7 (41%)	38 (45%)
Prefer not to answer	0 (0%)	1 (4%)	0 (0%)	0 (0%)	1 (1%)
**CeAD risk influences…**					
Pretreatment screening*	25 (83%)	23 (85%)	4 (36%)	4 (24%)	56 (66%)
C-T SMT instruction*	17 (57%)	11 (41%)	0 (0%)	3 (18%)	31 (36%)
Full c-spine SMT instruction*	22 (73%)	24 (89%)	2 (18%)	4 (24%)	52 (61%)
Upper c-spine SMT instruction*	5 (17%)	2 (7%)	1 (9%)	4 (24%)	12 (14%)
Neither full nor upper c-spine*	2 (7%)	0 (0%)	7 (64%)	4 (24%)	13 (15%)

* Significant difference across belief categories (Association vs. Causal Link vs. Other) by Pearson chi-square test (*p* < 0.05). No Response category excluded from significance testing

Percentages in belief-category columns reflect the proportion of each belief group endorsing that row characteristic. Percentages in the Total column reflect the proportion of the full analytic sample (*N* = 85)

Abbreviations: CeAD, cervical artery dissection; C-T, cervicothoracic; cSMT, cervical spinal manipulative therapy; c-spine, cervical spine

Among the 76 respondents who answered Q10 (resource selection), a median of 3 resources were selected (mean = 2.87, range 0–7). Classroom faculty members selected a mean of 2.95 resources compared to 2.64 among clinical faculty members; this difference was not statistically significant (Mann-Whitney U = 781.5, *p* = 0.338). Systematic reviews and meta-analyses were the most frequently endorsed resource in both classroom (57%) and clinical (58%) settings, while endorsement of individual resources did not differ significantly between classroom and clinical faculty, or between faculty members endorsing an associational vs. causal view of the cSMT–CeAD relationship. (Table 3)

**Table 3:** Knowledge resource endorsement by teaching setting among faculty who answered Q7 (*n* = 76)

Resource	Classroom (*n* = 42)	Clinical (*n* = 33)	*p*-value
Systematic reviews and meta-analyses	24 (57%)	19 (58%)	1.000
Personal/clinical experience	25 (60%)	14 (42%)	0.167
Anatomic/cadaveric studies	19 (45%)	14 (42%)	0.820
Case reports	19 (45%)	11 (33%)	0.348
Expert opinion of chiropractic educators	17 (40%)	10 (30%)	0.469
Clinical experience of colleagues	15 (36%)	13 (39%)	0.812
Narrative reviews	5 (12%)	6 (18%)	0.520

All comparisons by Fisher’s exact test. No significant differences were observed between classroom and clinical faculty for any individual resource type (all *p* > 0.10)

### Qualitative results

**Fig. 1:**
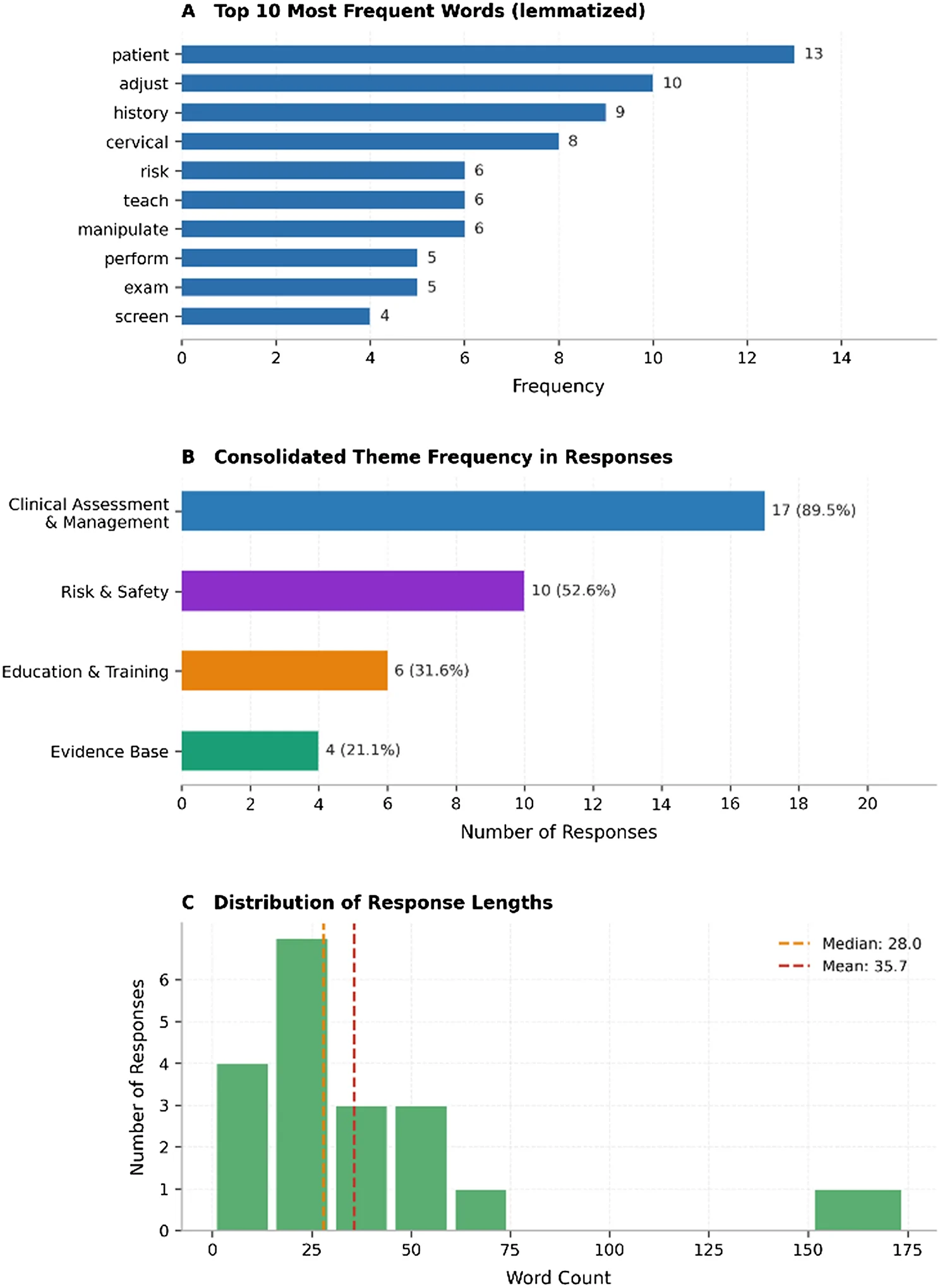
Free-text response analysis: (**A**) word frequency, (**B**) thematic distribution, and (**C**) response length

Analysis of free-text responses (*N* = 19; 12 classroom, 7 clinical) identified “patient,” “adjust,” and “history” as the most frequently occurring words after lemmatization. (Fig. 1, **Panel A**). Keyword-based thematic analysis across four consolidated themes revealed *Clinical Assessment & Management* as the dominant theme, present in 89.5% of responses and encompassing patient evaluation, screening protocols, and clinical decision-making (Fig. 1, **Panel B**). This prevalence reflects a shared faculty preoccupation with the practical clinical encounter – specifically, the acts of screening, history-taking and identifying risk factors – regardless of how individual faculty members conceptualized the underlying causal relationship. *Risk & Safety* appeared in 52.6% of responses and was notably characterized by individual faculty members’ use of clinically specific language (e.g., “warning signs”, “emergency situation”, “contraindications”) beyond the broader terms captured by the keyword dictionary, suggesting that faculty concern for patient safety is expressed in more operationally specific terms than those currently indexed. *Education & Training* (31.6%) and *Evidence Base* (21.1%) were less prevalent, though responses within the latter theme reflected meaningful uncertainty about the evidentiary basis for instructional decisions. Representative keywords and survey responses for each theme are presented in Table 4.

**Table 4:** Thematic analysis keywords and example responses

Theme	Keywords	Sample Response
Clinical Assessment & Management	patient, history, cervical, screening, manipulate, adjust, exam, perform	“We review contraindications to HVLA (relevant recent trauma, vascular pathology, signs of instability, connective tissue disorder). If a patient has acute new neck pain and or headaches we take a thorough history looking for any symptoms of neuro involvement…”
Risk & Safety	risk, CeAD, dissection, stroke, refer, vascular	“Almost 100% of the CAD risk is in the patient history and not hearing the patient describe how their symptoms came on. Students need hear these warning signs and know when to refer out to stabilize an emergency situation before adjusting them.”
Education & Training	teach, students, education, training	*“I am still dissatisfied with how this is taught and would appreciate having an exhaustive search on this topic clarify how we should all collectively teach this issue.”*
Evidence Base	evidence, research, literature, review, association	*“Much of my discussion involves a discussion of appropriate history and physical exam. An association between manipulation and CeAD certainly exists*,* but I have not been exposed to robust support indicating a particular style of SMT or refrainment (limiting rot*,* ext*,* etc.) has any particular influence.”*

## Discussion

Chiropractic educators and the programs in which they teach serve as a critical foundation for knowledge dissemination, shaping the educational experiences of both current students and practicing clinicians. Faculty are responsible for remaining current with scientific literature, particularly regarding the techniques, efficacy, and safety of chiropractic care, and as such represent a valuable source of knowledge into modern chiropractic thought and the curricular content informing clinical practice [13]. However, scant literature examines how chiropractic educators address specific clinical controversies within their curricular content. This study explored individual faculty members’ perspectives on the potential association between cSMT and CeAD and assessed how their attitudes may influence the way the topic is presented in chiropractic education.

Our analysis revealed that respondents teaching in clinical settings were nearly twice as likely as their classroom-based colleagues to perceive the cSMT – CeAD relationship as causal. In contrast, classroom faculty members more frequently characterized the relationship as associational – that is, rarely or never causal. Although the data did not provide direct explanations for this discrepancy, distinct contextual factors within each teaching setting may contribute to differing perceptions. Classroom faculty members may rely more heavily on literature casting doubt on a causal relationship [14–18] and may be less likely to engage in active clinical practice. Clinical faculty members, by contrast, are actively managing patient risk while supervising clinical interns, placing them in a position where medicolegal concerns and direct patient safety responsibilities may heighten caution. Notably, despite these divergent views on causation, both groups reported equal influence of CeAD risk on pretreatment screening instruction, suggesting that regardless of how individual faculty members conceptualize the underlying mechanism, they converge on the practical importance of thorough patient evaluation. Nevertheless, students may encounter differing messages about cSMT safety across classroom and clinical settings. Because faculty beliefs can shape student attitudes that persist into practice, such inconsistencies may have meaningful downstream consequences for how graduating clinicians approach cSMT safety [19]. Further work should aim to identify the underlying factors shaping these differing perceptions among faculty subgroups.

Systematic reviews and meta-analyses were the most endorsed knowledge sources informing a faculty member’s beliefs about technique modification and CeAD risk, at nearly identical rates across teaching settings and beliefs about the cSMT–CeAD association. The frequent endorsement of personal and clinical experience, particularly among classroom faculty, suggests that the same evidence may be interpreted through different clinical lenses. This may partly explain the inconsistencies in teaching strategies observed elsewhere in this study.

Many respondents reported modifying cervical spine adjusting techniques by minimizing rotation and extension, with a majority (61%) indicating they deliberately avoided these movements and emphasized this approach in their instruction. This adaptation may be informed by concerns about mechanical stress on the V3 segment of the vertebral artery, particularly near the C1 level [1, 7]. Notably, these modifications appear to extend beyond upper cervical techniques to other cervical regions, with CeAD risk influencing cervicothoracic SMT instruction across groups. This broader application is plausible given that multi-segmental motion has been detected several spinal segments distant from the manipulation contact point [20], despite the expectation that short-lever techniques produce localized forces [21]. Individual faculty may therefore be applying precautionary modifications across the cervical spine as a pragmatic response to this biomechanical uncertainty.

Thorough pretreatment screening, including a detailed history and physical exam, is essential in assessing a patient’s suitability for cSMT. In this study, individual faculty members who endorsed a causal link between CeAD and cSMT were more likely to modify their screening practices, although most respondents across groups recommended some form of CeAD-related screening. While evidence of a causal relationship remains inconclusive, clinicians must remain vigilant for concerning symptoms (e.g., atypical headache, neck pain, dizziness/vertigo) [22] in the context of a medical history of conditions with known associations with CeAD (e.g., migraine headache, hypertension, genetic connective tissue disorders, recent trauma) [22–24]. Recent work by Trager et al. characterizes symptoms associated with CeAD particular to chiropractic settings, however it should be noted that only 10 patients were ultimately included in this meta-analysis [25].

The dominance of the *Clinical Assessment & Management* theme is consistent with the finding that most respondents reported CeAD concerns influencing pretreatment screening instruction. This pattern extends beyond chiropractic education; Mourad et al. found that 70% of Italian physiotherapists reported performing additional screening prior to upper cervical manipulation, with safety perceptions varying significantly by spinal region [26]. Together, these findings suggest that heightened clinical vigilance around the cervical spine is a shared concern across professions that deliver spinal manipulation as well as patients who receive it [27].

Our findings further suggest that chiropractic faculty members are actively modifying teaching strategies specific to cSMT based on perceived CeAD risk. These modifications appear to be driven more by clinical experience and precautionary instinct than by engagement with published evidence. Bridging this gap through stronger integration of evidence-based content into CeAD-related instruction and more consistent educational frameworks across programs represents an important next step. Future research with larger, more diverse samples and more targeted questions regarding the basis for individual faculty’s beliefs could further clarify the drivers of cSMT-related instructional practices.

### Limitations

This study has several notable limitations. The sampling approach precluded calculation of an individual-level response rate, and while 17 of 28 invited programs participated, generalizability remains limited. Additionally, this study surveyed only English-speaking programs. This topic could benefit from a more international approach that includes more languages. No identifiable information was collected, precluding analysis of demographic factors that may influence faculty perspectives. The gatekeeper-led sampling approach did not preclude an individual from submitting multiple responses. The qualitative component comprised 19 free-text responses (25%) of variable length, and the cross-sectional design captures perspectives at a single point in time. Survey items assessing faculty beliefs did not distinguish between epidemiologic association, biological plausibility, and causal inference; derived belief categories should be interpreted as exploratory groupings rather than validated constructs. The survey assessed whether faculty modify instruction in response to CeAD risk but not the basis for those modifications, which may reflect evidence of engagement, medicolegal concerns, institutional expectations, or some combination. These bivariate tests assess statistical significance but neither estimate the magnitude of association nor adjust for potential confounders; the reported associations should be interpreted as unadjusted and preliminary. Finally, selection bias, social desirability bias, and instrument development bias (despite pilot review), cannot be excluded. Faculty with stronger views on patient safety or the cSMT–CeAD relationship may have been more likely to respond, and respondents may have overstated screening and precautionary practices to align with perceived professional expectations rather than their actual teaching practices. Although the instrument underwent face-validity review by four independent faculty members, its development by a single experienced chiropractic educator may have introduced assumptions reflecting the developer’s own professional perspective. Finally, reported beliefs may reflect variation in respondents’ recall, interpretation, and appraisal of the literature rather than a standardized assessment of the evidence.

## Conclusions

This study demonstrates that chiropractic faculty beliefs about the cSMT – CeAD relationship differ by instructional setting, with clinical faculty more likely to endorse a causal relationship and classroom faculty more likely to view the association as rarely or never causal. Whether these findings reflect differential exposure to adverse clinical events, varying engagement with the evidence base, or other factors warrants further investigation. The findings highlight a need for evidence-aligned curricular guidance to promote consistency in how CeAD-related risk is communicated and taught across chiropractic educational programs.

## Appendix A

You are being asked to take part in a research study about chiropractic faculty approaches to teaching cervical manipulation and the potential risk of cervical artery dissection (CeAD). Survey responses will remain anonymous and will not be separated by institution.

Your participation in this survey is voluntary and you may refuse to take part in the survey at any time without penalty. You are free to decline to answer any particular question you do not wish to answer for any reason and there are no foreseeable risks or discomforts to taking this survey. Your survey answers will be stored in a password protected electronic format. Qualtrics does not collect identifying information and therefore your responses will remain anonymous.

Your participation in this research study involves filling out an electronic survey. This survey should take you 10–15 min to complete.

To begin taking the survey, please click the arrow.

1. Have you taught some form of hands-on cervical manipulation or adjusting in the past 5 years at a college or university? Y/N.

- If yes: survey continues -If no: survey ends.

2. Please select the setting in which you teach cervical adjusting:

- Clinical setting.
- Classroom setting.
- Prefer not to answer.

3. Is the reported correlation between cervical adjusting and cervical artery dissection a subject you discuss when teaching?

4. If yes, which of the following phrases best describes your expert opinion on this association?

- There is no association between cervical adjustments and CeAD.
- There is an association between cervical adjustments and CeAD, but it is never causal.
- There is an association between cervical adjustments and CeAD, but it is very rarely causal.
- There is a causal link between cervical adjustments and CeAD, and certain history and/or examination procedures can reduce the risk of this sequela.
- There is a causal link between cervical adjustments and CeAD, however no changes/selections made in history or examination procedures can reduce the risk of this sequela.
- There is a causal link between cervical adjustments and CeAD, and modification to manipulative technique may influence the risk of CeAD.
- There is a causal link between cervical adjustments and CeAD, however no modification to manipulative technique can influence the risk of CeAD.
- If no continue to question 5.

5. Does the risk of CeAD affect how you teach pretreatment procedures prior to adjusting?

6. If yes, which of the following descriptions most closely aligns with how you teach pretreatment procedures?

- Asking specific history questions or performing specific exam procedures which assess the likelihood of a CeAD already in progress.
- Asking specific history questions or performing specific exam procedures which assess the likelihood of an underlying condition which increases the risk of CeAD when the patient is adjusted.
- Asking specific history questions or performing specific exam procedures which assess the likelihood of an underlying condition which increases the risk of CeAD for which adjustments may be erroneously blamed.
- Other (please describe):
- If no continue to question 7.

7. If you believe modifications *to cervical adjusting technique* may (or may not) influence the risk of CeAD, which resources/knowledge sources would you say most strongly influence that belief? (choose all that apply)

- Personal/clinical experience.
- Clinical experience of colleagues.
- Expert opinion of chiropractic educators.
- Anatomic and/or cadaveric studies in the scientific literature.
- Case reports in the scientific literature.
- Narrative reviews of the literature.
- Systematic reviews and meta-analyses of the literature.
- Other (please specify):

8. Does the risk of CeAD affect how you teach adjusting technique of the upper cervical spine only or the entire cervical spine?

9. Upper cervical spine only.

- Which of the following descriptions most closely aligns with the modification(s) to adjusting technique of the *upper cervical spine* when you teach?
  - Minimizing rotation in the upper cervical spine when performing adjustive thrusts.
  - Minimizing extension in the upper cervical spine when performing adjustive thrusts.
  - Minimizing lateral flexion/bending in the upper cervical spine when performing adjustive thrusts.
  - Minimizing the combination of rotation AND extension in the upper cervical spine when performing adjustive thrusts.
  - Minimizing the combination of rotation AND lateral flexion/bending in the upper cervical spine when performing adjustive thrusts.
  - Minimizing the combination of lateral flexion/bending AND extension in the upper cervical spine when performing adjustive thrusts.
  - Other (please describe):

10. Entire cervical spine.

- Which of the following descriptions most closely aligns with the modification(s) to adjusting technique of the *entire cervical spine* when you teach?
- Minimizing rotation in the cervical spine when performing adjustive thrusts.
- Minimizing extension in the cervical spine when performing adjustive thrusts.
- Minimizing lateral flexion/bending in the cervical spine when performing adjustive thrusts.
- Minimizing the combination of rotation AND extension in the cervical spine when performing adjustive thrusts.
- Minimizing the combination of rotation AND lateral flexion/bending in the cervical spine when performing adjustive thrusts.
- Minimizing the combination of lateral flexion/bending AND extension in the cervical spine when performing adjustive thrusts.
- Other (please describe):
- Neither.
  - Continue to question 11.

11. Does the risk of CeAD affect how you teach adjusting technique of the *cervicothoracic spine*?

- If yes, which of the following descriptions most closely aligns with the modification(s) to adjusting technique of the *cervicothoracic spine* when you teach?
  - Minimizing rotation in the cervicothoracic spine when performing adjustive thrusts.
  - Minimizing extension in the cervicothoracic spine when performing adjustive thrusts.
  - Minimizing lateral flexion/bending in the cervicothoracic spine when performing adjustive thrusts.
  - Minimizing the combination of rotation AND extension in the cervical and/or cervicothoracic spine when performing adjustive thrusts.
  - Minimizing the combination of rotation AND lateral flexion/bending in the cervical and/or cervicothoracic spine when performing adjustive thrusts.
  - Minimizing the combination of lateral flexion/bending AND extension in the cervical and/or cervicothoracic spine when performing adjustive thrusts.
  - Other (please describe):
- If no, continue to question 12.

12. Is there anything else you would like to describe regarding your approach to teaching of chiropractic technique and CeAD?

## Appendix B

**Table:** Checklist for Reporting Of Survey Studies (CROSS)

Section/topic	Item	Item description	Reported on page #
**Title and abstract**			
Title and abstract	1a	State the word “survey” along with a commonly used term in title or abstract to introduce the study’s design.	Title
	1b	Provide an informative summary in the abstract, covering background, objectives, methods, findings/results, interpretation/discussion, and conclusions.	Pg 3-4
Introduction			
Background	2	Provide a background about the rationale of study, what has been previously done, and why this survey is needed.	Pg 4-5
Purpose/aim	3	Identify specific purposes, aims, goals, or objectives of the study.	Pg 5
**Methods**			
Study design	4	Specify the study design in the methods section with a commonly used term (e.g., cross-sectional or longitudinal).	Pg 6
	5a	Describe the questionnaire (e.g., number of sections, number of questions, number and names of instruments used).	Pg 6
Data collection methods	5b	Describe all questionnaire instruments that were used in the survey to measure particular concepts. Report target population, reported validity and reliability information, scoring/classification procedure, and reference links (if any).	Pg 6
	5c	Provide information on pretesting of the questionnaire, if performed (in the article or in an online supplement). Report the method of pretesting, number of times questionnaire was pre-tested, number and demographics of participants used for pretesting, and the level of similarity of demographics between pre-testing participants and sample population.	N/A
	5d	Questionnaire if possible, should be fully provided (in the article, or as appendices or as an online supplement).	App. A
Sample characteristics	6a	Describe the study population (i.e., background, locations, eligibility criteria for participant inclusion in survey, exclusion criteria).	Pg 6
	6b	Describe the sampling techniques used (e.g., single stage or multistage sampling, simple random sampling, stratified sampling, cluster sampling, convenience sampling). Specify the locations of sample participants whenever clustered sampling was applied.	Pg 6
	6c	Provide information on sample size, along with details of sample size calculation.	Pg 6
	6d	Describe how representative the sample is of the study population (or target population if possible), particularly for population-based surveys.	Pg 6
Surveyadministration	7a	Provide information on modes of questionnaire administration, including the type and number of contacts, the location where the survey was conducted (e.g., outpatient room or by use of online tools, such as SurveyMonkey).	Pg 6
	7b	Provide information of survey’s time frame, such as periods of recruitment, exposure, and follow-up days.	Pg 6
	7c	Provide information on the entry process:–>For non-web-based surveys, provide approaches to minimize human error in data entry.–>For web-based surveys, provide approaches to prevent “multiple participation” of participants.	N/A
Study preparation	8	Describe any preparation process before conducting the survey (e.g., interviewers’ training process, advertising the survey).	N/A
Ethical considerations	9a	Provide information on ethical approval for the survey if obtained, including informed consent, institutional review board [IRB] approval, Helsinki declaration, and good clinical practice [GCP] declaration (as appropriate).	Pg 6
	9b	Provide information about survey anonymity and confidentiality and describe what mechanisms were used to protect unauthorized access.	Pg 6
Statisticalanalysis	10a	Describe statistical methods and analytical approach. Report the statistical software that was used for data analysis.	Pg 6
	10b	Report any modification of variables used in the analysis, along with reference (if available).	Pg 7-8
	10c	Report details about how missing data was handled. Include rate of missing items, missing data mechanism (i.e., missing completely at random [MCAR], missing at random [MAR] or missing not at random [MNAR]) and methods used to deal with missing data (e.g., multiple imputation).	Pg 7, Tables 1,2
	10d	State how non-response error was addressed.	Pg 7
	10e	For longitudinal surveys, state how loss to follow-up was addressed.	N/A
	10f	Indicate whether any methods such as weighting of items or propensity scores have been used to adjust for non-representativeness of the sample.	N/A
	10g	Describe any sensitivity analysis conducted.	N/A
**Results**			
Respondent characteristics	11a	Report numbers of individuals at each stage of the study. Consider using a flow diagram, if possible.	Pg 7
	11b	Provide reasons for non-participation at each stage, if possible.	N/A
	11c	Report response rate, present the definition of response rate or the formula used to calculate response rate.	Pg 7
	11d	Provide information to define how unique visitors are determined. Report number of unique visitors along with relevant proportions (e.g., view proportion, participation proportion, completion proportion).	N/A
Descriptiveresults	12	Provide characteristics of study participants, as well as information on potential confounders and assessed outcomes.	Fig 1
Main findings	13a	Give unadjusted estimates and, if applicable, confounder-adjusted estimates along with 95% confidence intervals and p-values.	Pg 8-9
	13b	For multivariable analysis, provide information on the model building process, model fit statistics, and model assumptions (as appropriate).	N/A
	13c	Provide details about any sensitivity analysis performed. If there are considerable amount of missing data, report sensitivity analyses comparing the results of complete cases with that of the imputed dataset (if possible).	N/A
**Discussion**			
Limitations	14	Discuss the limitations of the study, considering sources of potential biases and imprecisions, such as non-representativeness of sample, study design, important uncontrolled confounders.	Pg 15
Interpretations	15	Give a cautious overall interpretation of results, based on potential biases and imprecisions and suggest areas for future research.	Pg 11-13
Generalizability	16	Discuss the external validity of the results.	Pg 14
**Other sections**			
Role of funding source	17	State whether any funding organization has had any roles in the survey’s design, implementation, and analysis.	Pg. 17
Conflict of interest	18	Declare any potential conflict of interest.	Pg. 16
Acknowledgements	19	Provide names of organizations/persons that are acknowledged along with their contribution to the research.	Title Page and pg 17

## Supplementary Information

Below is the link to the electronic supplementary material.


Supplementary Material 1


## Data Availability

The raw data supporting the findings of this study are available from the corresponding author upon reasonable request.

## Abbreviations

CeAD: Cervical artery dissection
cSMT: Cervical spinal manipulative therapy
V3: Segment 3 of the vertebral artery
C1: Cervical vertebrae 1
C2: Cervical vertebrae 2
